# TBX3 represses TBX2 under the control of the PRC2 complex in skeletal muscle and rhabdomyosarcoma

**DOI:** 10.1038/s41389-019-0137-z

**Published:** 2019-04-12

**Authors:** Teak-Jung Oh, Abhinav Adhikari, Trefa Mohamad, Aiysha Althobaiti, Judith Davie

**Affiliations:** 1 School of Molecular and Cellular Biology, University of Illinois at Urbana-Champaign, Urbana, IL 61801 USA; 2 Department of Biochemistry and Molecular Biology and Simmons Cancer Institute, Southern Illinois University School of Medicine, Carbondale, IL 62901 USA; 3 Biology Department, College of Science, Salahaddin University, Erbil, Kurdistan 44002 Iraq

## Abstract

TBX2 and TBX3 function as repressors and are frequently implicated in oncogenesis. We have shown that TBX2 represses p21, p14/19, and PTEN in rhabdomyosarcoma (RMS) and skeletal muscle but the function and regulation of TBX3 were unclear. We show that TBX3 directly represses TBX2 in RMS and skeletal muscle. TBX3 overexpression impairs cell growth and migration and we show that TBX3 is directly repressed by the polycomb repressive complex 2 (PRC2), which methylates histone H3 lysine 27 (H3K27^me^). We found that TBX3 promotes differentiation only in the presence of early growth response factor 1 (EGR1), which is differentially expressed in RMS and is also a target of the PRC2 complex. The potent regulation axis revealed in this work provides novel insight into the effects of the PRC2 complex in normal cells and RMS and further supports the therapeutic value of targeting of PRC2 in RMS.

## Introduction

Rhabdomyosarcoma (RMS) is the most common soft tissue pediatric sarcoma, which is thought to largely arise from the skeletal muscle lineage^1^. The more common form of the disease is the embryonal subtype (ERMS), characterized by loss of heterozygosity at the *11p15* locus, a region which harbors insulin-like growth factor 2. Alveolar RMS (ARMS) is the more aggressive form of RMS that is characterized by t(2;13)(q35;q14) or t(1;13)(q36;q14) translocations. The translocations result in chimeric transcripts that fuse the 5′ portion of the paired box proteins 3 or 7 (PAX3 or PAX7), including an intact DNA-binding domain, to the transactivation domain of a forkhead transcription factor (FKHR), creating novel PAX3-FKHR (t(2;13)(q35;q14)) or PAX7-FKHR (t(1;13)(q36;q14)) fusion proteins^2,3^. RMS is diagnosed by observation of unique skeletal muscle cell morphology phenotypes and the presence of myogenic markers such as myogenic regulatory factors (MRFs)^4^, yet these factors appear to be inactive in RMS^5^.

The T-box family of transcription factors are highly conserved and related throughout all metazoan lineages. They share a common DNA-binding domain known as the T-box motif and participate in diverse types of organogenesis and developmental regulation^6^. The T-box motif binds to the core sequence GGTGTGA known as the T-element^7^. Distinct from most members of the T-box family, TBX2 is known as a potent transcriptional repressor that functions in both embryonic development, and if deregulated, tumorigenesis^8^. The oncogenic potential of TBX2 was first identified by its ability to bypass cellular senescence in a *Bmi* ^−/−^ mouse embryo fibroblast model^9^. TBX2 has been shown to promote cell proliferation and block cellular senescence by repressing *CDKN1A* (p21), *CDKN2A* (p14/19^ARF^)^10^, and *PTEN*^11^.

Another member of the T-box family, TBX3, is also characterized as a transcriptional repressor and developmental regulator^12^. TBX2 and TBX3 are highly related and thought to have arisen from a gene duplication event^13^. In the case of deregulation, TBX3 also acquires the potential to become an oncogene in melanoma cells^14^. TBX3 has been found to be overexpressed in melanoma^15^ and several sarcomas^16^. TBX3 has been shown to promote tumor metastasis and the migration of breast cancer cells^17^. In MCF-12A breast epithelial cells and B16 mouse melanoma cells, TBX3 has been shown to directly repress TBX2 under the control of TGF-β1^18^. Our lab has shown that TBX3 is expressed throughout skeletal muscle differentiation, whereas the expression of TBX3 in RMS cell lines is almost completely abrogated^11^.

The polycomb repressive complexes 1 and 2 (PRC1/2) are critical in the precise and accurate regulation of development in many physiological systems including skeletal muscle^19^. PRC1 and PRC2 work synergistically and indispensably together for transcriptional repression of target gene by ubiquitylation of histone H2A lysine 119 residue and methylation of histone 3 lysine 27 (H3K27) residues, respectively^20^. The catalytic subunit of PRC2 is *enhancer of zeste 2* (*Ezh2*)^21^.

Many studies have revealed that EZH2 is overexpressed in a large number of cancers^22^. In RMS, pharmacological inhibition of EZH2 in ERMS resulted in reduced proliferation and pharmacological inhibition or depletion of EZH2-induced myogenic differentiation in these cells^23^. EZH2 depletion in ARMS has also been shown to inhibit proliferation and initiate apoptosis^24^. JARID2, a founding member of Jumonji family of proteins, is known to be a substoichiometric component of PRC2 that appears to function in targeting PRC2 activity^25^. JARID2 is highly expressed in ARMS and contributes to the inhibition of differentiation in ARMS cells^26^.

In this work, we show that TBX3 represses TBX2 under the control of the PRC2 complex in RMS and skeletal muscle. RMS cells and proliferating skeletal muscle cells contain high levels of EZH2 that represses TBX3 and allows TBX2 expression. Depletion of EZH2 upregulates TBX3 which then down regulates TBX2. We also show that the early growth response gene *EGR1* is correlated with the induction of differentiation and repressed by PRC2 in ARMS. Discovery of this novel PRC2-TBX3-TBX2 genetic axis has important implications for understanding the mechanisms that drive proliferation and differentiation in RMS and skeletal muscle.

## Results

### TBX3 represses TBX2

**Fig. 1:**
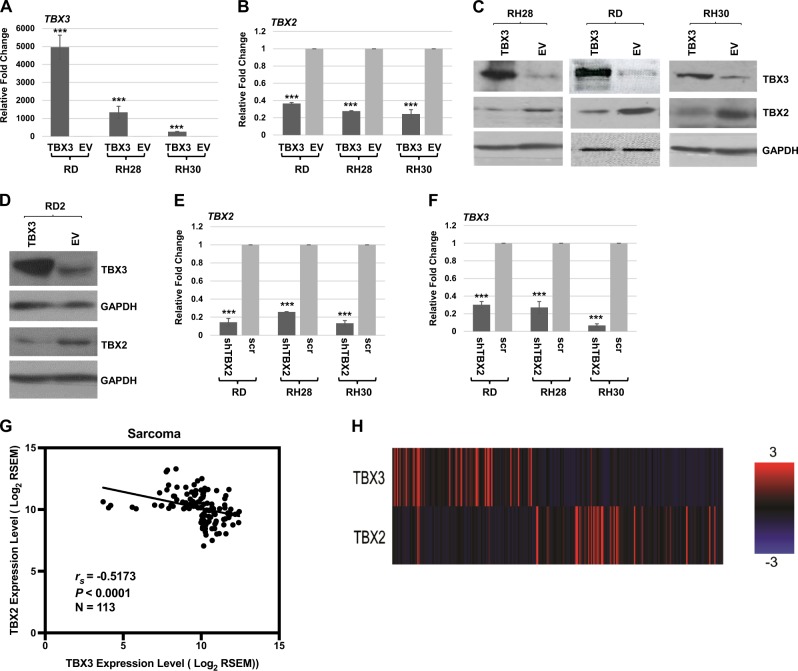
TBX3 represses TBX2 in RMS. **a**, **b** The expression construct pEF-TBX3 (TBX3) or pEF empty vector (EV) was transiently transfected into RD, RH28, and RH30 cell lines and assayed by qRT-PCR using primers against *TBX3* (**a**) and *TBX2* (**b**). Error bars, standard errors (S.E.) and ***p ≤ 0.001 versus EV. **c** Protein extracts from the transiently transfected isolates in (**a**) were used for western blot analysis with antibodies against the indicated proteins. **d** pEF-TBX3 (TBX3) and pEF (EV) were transiently transfected into RD2 cells and examined by western bot analysis against the indicated proteins. GAPDH was used as the loading control. **e**, **f** A short-hairpin RNA construct against TBX2 (shTBX2) or a scrambled control (scr) construct was transiently transfected into RD, RH28, and RH30 cell lines and assayed by qRT-PCR using primers against *TBX2* (**e**) and *TBX3* (**f**). Error bars, S.E. and ***p ≤ 0.001 versus EV. TBX3 and TBX2 are inversely correlated in sarcoma. (**g**) Scatter plot representation of mRNA expression of *TBX3* and *TBX2* in sarcoma patients from The Cancer Genome Atlas (TCGA) (*r*_*s*_ = −0.5173, *p* < 0.0001, *N* = 113). (**h**) mRNA expression data for TBX3 and TBX2 in sarcoma patients were clustered and downloaded using cBioPortal (*N* = 263). Red represents higher expression while blue represents decreased expression

We have previously shown that TBX2 is highly expressed in RMS while TBX3 is not^11,27^. In skeletal muscle, TBX2 is expressed in proliferating myoblasts, but sharply downregulated upon differentiation while TBX3 is expressed throughout myogenesis and highly expressed during differentiation^11,27^. To understand the potential role of TBX3 in RMS, we transiently transfected RMS cell lines representing both ERMS (RD and RD2) and ARMS (RH30 and RH28) with an expression plasmid for TBX3^11^. As anticipated, we observed that TBX3 was upregulated (Fig. 1a). Upon the upregulation of TBX3, we found that TBX2 was downregulated (Fig. 1b) in RH30, RH28, and RD cells. The degree of TBX3 overexpression in RMS cells corresponded to the degree of TBX2 repression in each cell line tested (Fig. 1a, b). The repression of TBX2 by TBX3 was confirmed at the protein level in RD, RH28, and RH30 cell lines (Fig. 1c). For the RD2 cell line, RNA results were inconsistent but the protein analysis confirmed that TBX3 repression of TBX2 could be observed in these cells as well (Fig. 1d).

To understand if TBX2 and TBX3 reciprocally repressed each other’s expression, we asked if TBX2 could also repress TBX3. RMS cell lines were transiently transfected with a shRNA construct against TBX2^27^ and assayed for expression of TBX2 and TBX3. As anticipated, we observed that TBX2 was depleted (Fig. 1e), but saw no upregulation of TBX3 (Fig. 1f). Thus, depletion of TBX2 does not upregulate TBX3. In fact, TBX3 was even more strongly downregulated in the absence of TBX2, indicating that TBX2 may play a role in activating TBX3 in RMS. Our data showed that TBX3 represses TBX2 in RMS cell lines so we asked if this expression correlation could also be observed in additional sarcomas using available patient data. Sarcoma expression data for TBX3 and TBX2 were downloaded from The Cancer Genome Atlas (TCGA) (https://cancergenome.nih.gov/) database using GDAC Firehose. Two cohorts of the top one-third TBX3 and TBX2 expressing samples were combined and duplicates samples were removed. A correlation analysis was performed and a scatter plot was generated for TBX3 and TBX2 expression (Fig. 1g). TBX2 and TBX3 were found to be inversely correlated (Spearman correlation coefficient (*r*_*s*_) = −0.5173, *p* < 0.0001, *N* = 113), which was also evident from two distinct clusters on the heatmap with opposite expression (*N* = 263) (Fig. 1h).

**Fig. 2:**
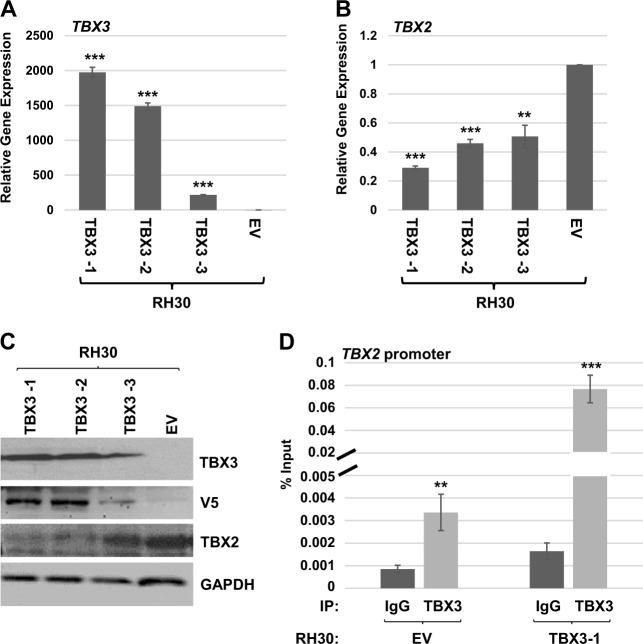
TBX3 directly represses TBX2 in RH30 cells. **a** pEF-TBX3 (TBX3) or pEF-empty vector (EV) was stably transfected into RH30 cells and the expression of *TBX3* in three independent isolates was assayed by qRT-PCR analysis. Error bars, S.E. and ***p ≤ 0.001 versus EV. **b** Expression of *TBX2* in the cells described in (**a**) was measured by qRT-PCR analysis. Error bars, S.E. and **p ≤ 0.01, ***p ≤ 0.001 versus EV. **c** Protein extracts from the stably transfected isolates in (**a**) were used for western blot analysis with antibodies against the indicated proteins. GAPDH was used as a loading control. **d** TBX3 binds to the *TBX2* promoter. ChIP assays were performed on RH30 EV and RH30 TBX3-1 cells with antibodies against TBX3 or IgG and primers to the *TBX2* promoter. Error bars, S.E. and **p ≤ 0.01, ***p ≤ 0.001 versus IgG

To characterize the cellular effects of TBX3 in RMS, we generated stable RH30 cell lines expressing TBX3 and three independent clonal isolates were used for further analysis. Expression of exogenous TBX3 was confirmed at both the mRNA level (Fig. 2a) and the protein level (Fig. 2c). We found that stable overexpression of TBX3 resulted in high levels of *TBX3* mRNA expression (Fig. 2a) and repressed *TBX2* mRNA expression (Fig. 2b). The repression of TBX2 by TBX3 could also be observed at the protein level (Fig. 2c). TBX3 was detected with antibodies against TBX3 and the V5 epitope tag only present on exogenous TBX3. In each case, the degree of overexpressed TBX3 correlated to the degree of TBX2 repression, confirming that TBX3 represses TBX2.

To determine if the repression of TBX2 is directly mediated by TBX3, chromatin immunoprecipitation (ChIP) assays were performed using antibodies against TBX3 and primers against the *TBX2* promoter and RH30 cells either overexpressing TBX3 or vector only. We found that TBX3 was enriched on the *TBX2* promoter, confirming that TBX3 directly represses the *TBX2* promoter (Fig. 2d). Intriguingly, this enrichment could be observed in both RH30 vectors, only cells that express low endogenous levels of TBX3 and in RH30 cells overexpressing TBX3 (Fig. 2d). Given the low level of endogenous TBX3 expression in RMS cells, it is not surprising that the degree of enrichment was greatly enhanced in the TBX3 overexpression cell line.

### TBX3 overexpression impairs proliferation, migration, and anchorage-independent growth of RH30 cells but does not promote differentiation

**Fig. 3:**
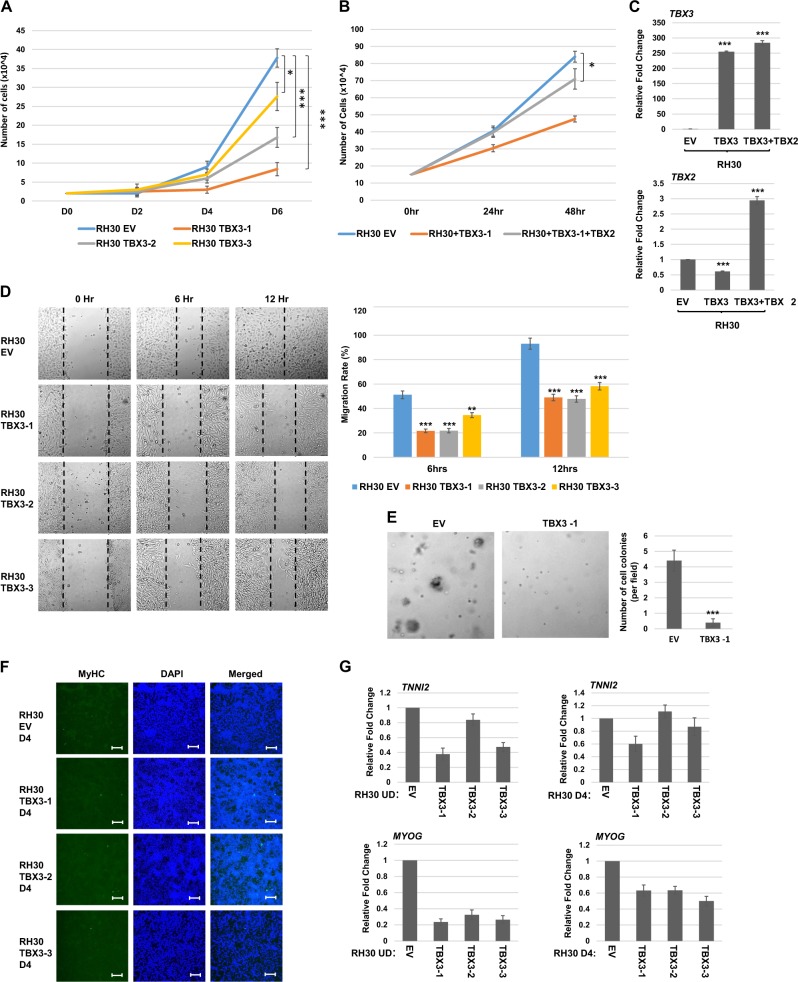
TBX3 inhibits oncogenic properties but does not promote differentiation of RH30 cells. **a** TBX3 inhibits proliferation. Identical number of cells were seeded and cells were counted every 2 days. Error bars, S.E. and *p ≤ 0.05 and ***p ≤ 0.001 versus EV. **b** TBX2 partially rescues the proliferation defect. The RH30 TBX3-1 cell line was transiently transfected with pEF TBX2 (TBX2) or pEF empty vector (EV) and assayed for proliferation as in (**a**) except cell counts were performed every 24 h. **c** mRNA expression of *TBX3* and *TBX2* in the cells shown in (**b**) was assayed by qRT-PCR. Error bars, S.E. and ***p ≤ 0.001 versus EV. **d** TBX3 inhibits migration. Scratch assays were performed on RH30 cells overexpressing TBX3 (TBX3) or pEF (EV). Images were taken at ×100 magnification. Migration rate is quantitated in the right panel. Error bars, S.E. and **p ≤ 0.01, ***p ≤ 0.001 versus EV. **e** Soft agar assays on RH30 TBX3-1 and EV cell lines. Images were taken at ×100 magnification and the number of colonies was quantified by counting in five random fields. Error bars, S.E. and ***p ≤ 0.001 versus EV. **f** RH30 overexpressing TBX3 cell lines were assayed by immunofluorescence with myosin heavy chain antibodies (MyHC) following 4 days of differentiation (D4). Images were taken at ×100 and scale bars represent 100 μm. DAPI was used to stain nuclei. **g** mRNA expression of differentiation specific genes is not upregulated in RH30 cells overexpressing TBX3 in the presence (D4) or absence (UD) of differentiation conditions as assayed by qRT-PCR. Error bars, S.E.

Previous work in the lab has shown that TBX2 plays a critical role in maintaining oncogenic properties such as proliferation, migration, and anchorage-independent growth in RMS^11,27^. TBX3 has been implicated in oncogenesis in breast cancer and melanoma^14,17^. To determine if TBX3 inhibited oncogenesis by repressing TBX2 or instead had a TBX2 independent role in promoting oncogenesis in RMS cells, we assayed for the growth properties of RH30 cells expressing exogenous TBX3. We found that proliferation was strongly inhibited by TBX3 and closely correlated with the degree of TBX3 expression in each clonal isolate (Fig. 3a). As TBX3 represses TBX2, which we showed to be required for proliferation^27^, we asked if restoration of TBX2 could reverse the effect of TBX3 overexpression. We found that proliferation was partially restored when TBX2 expression was rescued using TBX2 overexpression construct in TBX3 overexpressing RH30 cells (Fig. 3b). Intriguingly, these cells contained even higher levels of TBX2 than RH30 cells (Fig. 3c), yet proliferation was not fully rescued. These results strongly suggest that TBX3 does not independently promote proliferation in RMS cells and rather, functions to inhibit proliferation by repressing TBX2 and additional unknown mechanisms. The migration ability of RH30 cells stably overexpressing TBX3 was assayed by a scratch wound assay and we found that TBX3 also inhibited cell migration as assayed by wound recovery (Fig. 3d). The anchorage-independent growth of RH30 cells stably overexpressing TBX3 was examined by a soft agar assay and all cell lines showed an inhibition of anchorage-independent growth (Fig. 3e). Together, these results strongly suggest that TBX3 does not appear to confer oncogenic properties in RMS cells and appears instead to function as a tumor suppressor by repressing TBX2.

To investigate if overexpression of TBX3 in RH30 cells could promote differentiation as we observed with the depletion of TBX2^27^, we subjected the TBX3 overexpressing cells to differentiation conditions. The cell lines were then assayed for expression of myosin heavy chain (MyHC) by immunofluorescence. RH30 cells expressing exogenous TBX3 did not show the presence of MyHC positive cells (Fig. 3f). We also assayed for additional markers of differentiation such as *TROPONIN2* (*TNNI2*) and *MYOGENIN* (*MYOG*) in cells under normal growth conditions and under differentiation conditions. We found no upregulation of these markers under either condition (Fig. 3g). These data show that TBX3 expression in RH30 cells is not sufficient to induce myogenic differentiation.

### Overexpression of TBX3 impairs proliferation and migration and promotes differentiation of RD cells

**Fig. 4:**
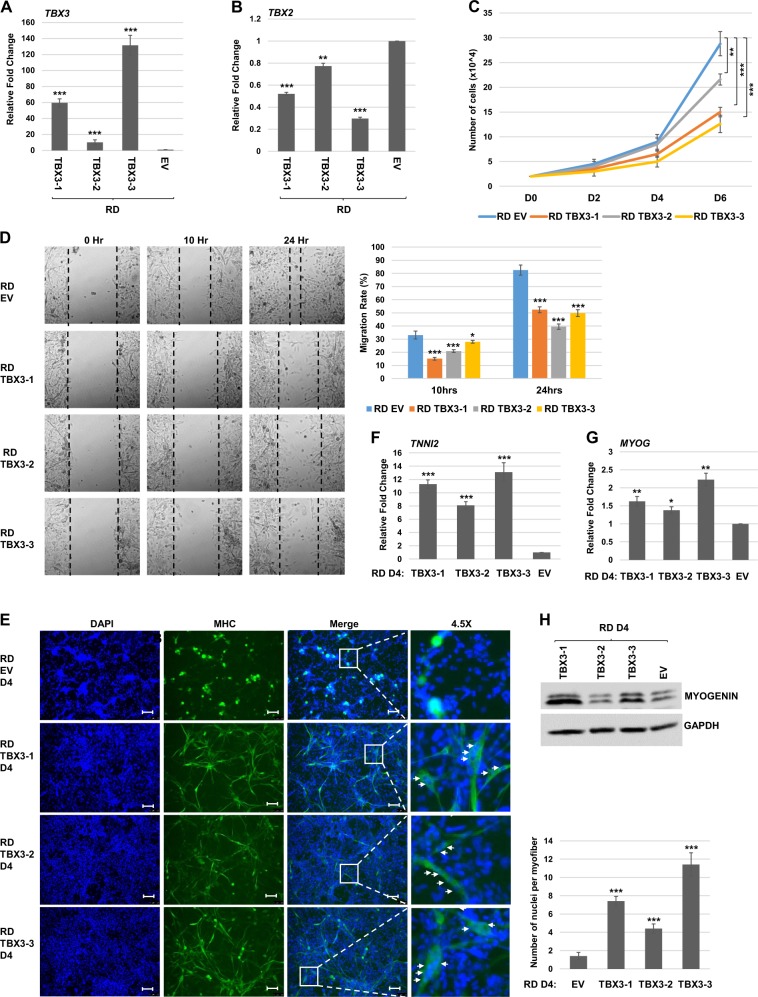
TBX3 inhibits proliferation and promotes differentiation in RD cells. **a**, **b** RD cells were transfected with pEF-TBX3 (TBX3) or pEF (EV) and three independent clones were characterized. mRNA expression of *TBX3* (**a**) and *TBX2* (**b**) was assayed by qRT-PCR. Error bars, S.E. and **p ≤ 0.01, ***p ≤ 0.001 versus EV. **c** TBX3 inhibits proliferation. RD cells overexpressing TBX3 shown in (**a**) were assayed for proliferation by a cell counting assay. Error bars, S.E. and **p ≤ 0.01, ***p ≤ 0.001 versus EV. **d** Scratch assays were performed on RH30 cells overexpressing TBX3 (TBX3) or pEF (EV). Images were taken at ×100 magnification. Migration rate is quantitated in the right panel. Error bars, S.E. and *p ≤ 0.05, ***p ≤ 0.001 versus EV. **e** RD cells overexpressing TBX3 were assayed by immunofluorescence with antibodies against MyHC after 4 days differentiation (D4) DAPI was used to stain nuclei. Images were taken at ×100 and scale bars represent 100 μm. Nuclei per myofiber are quantitated in the right panel. **f**, **g** mRNA expression of *TNNI2* (**f**) and *MYOG* (**g**) were examined in RD cells overexpressing TBX3 by qRT-PCR after 4 days of differentiation (D4). Error bars, S.E. and *p ≤ 0.05, **p ≤ 0.01, ***p ≤ 0.001 versus EV. **h** Protein extracts from RD cells as in (**f**, **g**) were used for western blot assays with antibodies against MYOG. GAPDH was used as a loading control

Our results suggested that TBX3 functions as a tumor suppressor in ARMS by repression of TBX2. To extend these finding to the ERMS subtype, we established stable cell lines overexpressing TBX3 in RD cells and characterized three independent clonal isolates. We found that exogenous *TBX3* mRNA was highly expressed in each cell line (Fig. 4a) and *TBX2* mRNA was repressed (Fig. 4b). Cell proliferation assays showed an impaired proliferation capability of these cells which correlated with the degree of TBX3 overexpression and TBX2 repression (Fig. 4c). Scratch wound assays also showed reduced migration in RD cells expressing exogenous TBX3 (Fig. 4d). Thus, TBX3 appears to function as an inhibitor of cell growth and migration in both ERMS and ARMS cells. To determine if TBX3 could promote differentiation in RD cells, cells overexpressing TBX3 were subjected to differentiation conditions. Cells were assayed for the expression of MyHC by immunofluorescence and we found that MyHC cells were observable when TBX3 was expressed (Fig. 4e). Not only was the presence of MyHC observed but multinucleate myotubes could also be observed upon TBX3 overexpression. When expression of differentiation specific genes including *TNNI2* (Fig. 4f) and *MYOG* (Fig. 4g) were examined, upregulation of these genes was observed. MYOG was also assayed at the protein level and the degree of MYOG upregulation closely correlated with the level of upregulated TBX3 (Fig. 4h). Together, these results show that TBX3 can induce differentiation in RD cells, in contrast to what was seen in RH30 cells.

### EZH2 is upregulated in RMS and targets TBX3

**Fig. 5:**
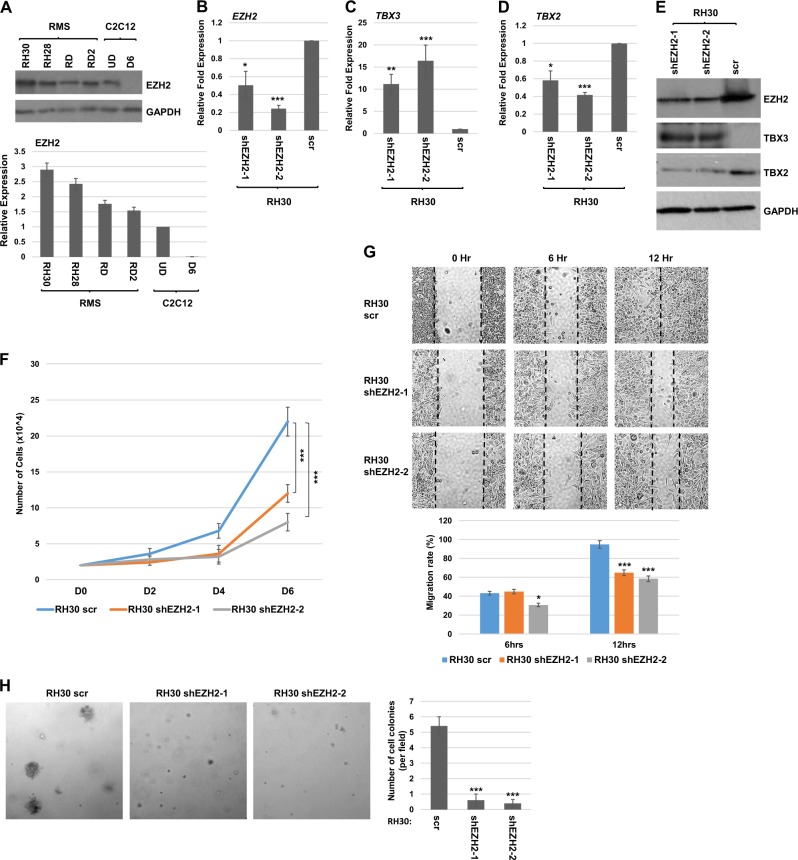
EZH2 is upregulated in RMS and targets TBX3. **a** Expression of EZH2 was examined by western blot assays for RMS cell lines and proliferating C2C12 cells (UD) and after 6 days of differentiation (D6). Replicate blots are quantitated in the lower panel. GAPDH was used as a loading control. **b**–**d** RH30 cells were transfected with shEZH2 and scrambled control (scr) and two independent clones were characterized following selection for stable isolates. Expression of *EZH2* (**b**), *TBX3* (**c**), and *TBX2* (**d**) were assayed by qRT-PCR. Error bars, S.E. and *p ≤ 0.05, **p ≤ 0.01, ***p ≤ 0.001 versus scr. **e** Protein extracts from the cells in (**b**) were used for western blot assays with antibodies against the indicated proteins. GAPDH was used as a loading control. **f** Depletion of EZH2 impairs proliferation. RH30 cells as in (**b**) were seeded with the same number of cells and harvested for cell counts every 2 days. Error bars, S.E. and ***p ≤ 0.001 versus scr. **g** Scratch assays were performed for the RH30 cells as in (**b**). Images were taken at ×100 magnification. Migration rate was quantitated in the lower panel. Error bars, S.E. and *p ≤ 0.05, ***p ≤ 0.001 versus scr. **h** Soft agar assays were performed on the cells as in (**b**). Images were taken at ×100 magnification and the number of colonies quantified by counting in five random fields. Error bars, S.E. and ***p ≤ 0.001 versus scr

As has been previously shown^23,24,26^, the catalytic subunit of the PRC2 complex, EZH2, is upregulated in RMS cell lines when compared to C2C12 cells (Fig. 5a). We asked if depletion of EZH2 in RH30 cells altered the expression of *TBX2* or *TBX3*. *EZH2* mRNA was depleted with a shRNA construct (shEZH2) in RH30 cells and stable cell lines expressing shEZH2 were selected. Two clones were further characterized and we noted that in both cell lines, EZH2 was modestly depleted at the level of mRNA (Fig. 5b) and protein (Fig. 5e). These results are in contrast to results obtained with a transient approach with siRNA against EZH2 in RH30 cells, which resulted in a severe downregulation of EZH2 and apoptosis^24^. Consistent with these results, we found that the cell lines recovered in our approach were only modestly depleted for EZH2. We found that *TBX3* mRNA was upregulated upon EZH2 depletion in RH30 cells (Fig. 5c). We then examined *TBX2* mRNA expression and found that *TBX2* mRNA was accordingly downregulated (Fig. 5d). These results were confirmed at the protein level as well (Fig. 5e).

To examine the effects of EZH2 depletion in RH30 cells, we examined cell growth properties of the cells. Cell proliferation assays showed that depletion of EZH2 inhibited RH30 cell growth (Fig. 5f). The migration and motility of RH30 cells were examined by a scratch assay and both of the EZH2 depletion lines showed slower scratch recovery than the control (Fig. 5g). Anchorage-independent growth was assayed by soft agar assays and showed that EZH2 depletion inhibited anchorage-independent growth (Fig. 5h). Thus, depletion of EZH2 inhibits proliferation, migration, and anchorage-independent growth of RH30 cells.

**Fig. 6:**
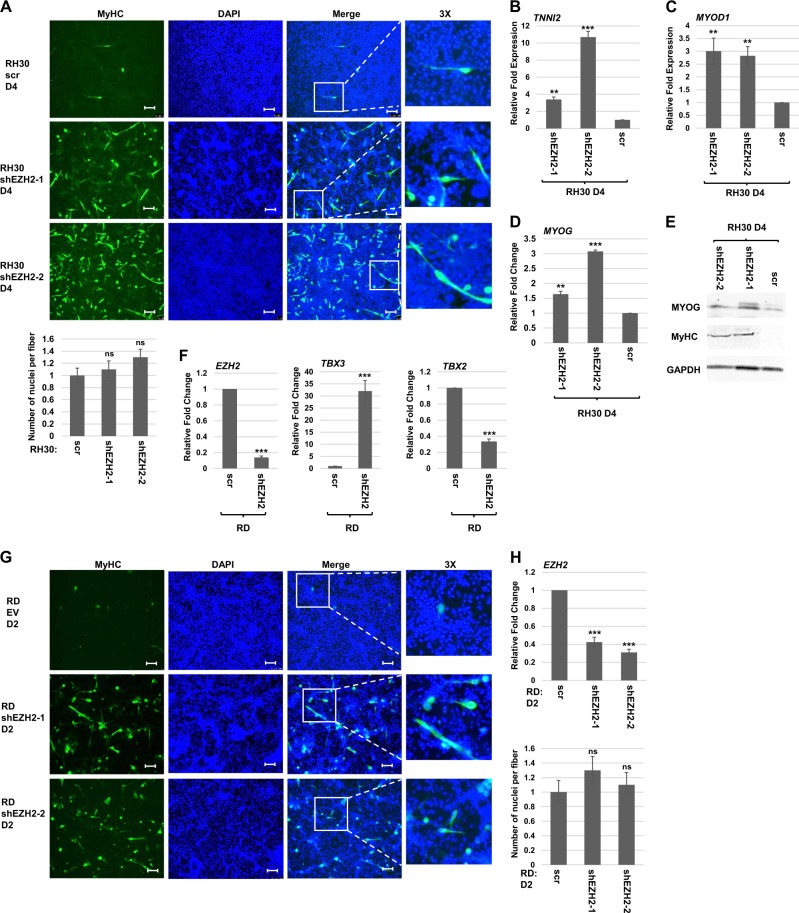
EZH2 represses TBX3 in RD cells and promotes differentiation of ARMS and ERMS cells. **a** RH30 shEZH2 cell lines were assayed by immunofluorescence with antibodies against MyHC after 4 days of differentiation. DAPI was used to stain the nuclei. Images were taken at ×100 and scale bars represent 100 μm. **b–d** The mRNA expression of *TNNI2* (**b**), *MYOD1* (**c**), and *MYOG* (**d**) were assayed in RH30 shEZH2 cell lines after 4 days of differentiation (D4) by qRT-PCR. Error bars, S.E. and **p ≤ 0.01, ***p ≤ 0.001 versus scr. **e** Protein extracts from RH30 cells as in (**b**) were used for western blot analysis with antibodies against the indicated proteins. GAPDH was used as the loading control. **f** EZH2 represses TBX3 in RD cells. RD cells were transiently transfected with shEZH2 and scr control and assayed by mRNA expression of the indicated genes by qRT-PCR. Error bars, S.E. and ***p ≤ 0.001 versus scr. **g** RD cells were transiently transfected with shEZH2 and scr control and assayed by immunofluorescence with antibodies against MyHC after 2 days of differentiation (D2). DAPI was used to stain nuclei. Images were taken at ×100 and scale bars represent 100 μm. Nuclei per myofiber are quantitated in the right panel. Error bars, S.E., and n.s. represent not statistically significant versus scr. **h** The mRNA expression of *EZH2* in the cells shown in (**g**) was assayed by qRT-PCR and shown in the right panel. Error bars, S.E. and ***p ≤ 0.001 versus scr

We next asked if depletion of EZH2 could promote differentiation in RH30 cells. No overt signs of differentiation were observed in the earlier siRNA approach which led to apoptosis^24^. In this prior study, cells were grown in growth medium. Here, RH30 cells modestly depleted for EZH2 were subject to differentiation conditions and examined for MyHC by immunofluorescence. We found that RH30 cells depleted for EZH2 showed MyHC expression, indicating that depletion of EZH2 could promote differentiation (Fig. 6a). We also found that mRNA expression of differentiation specific genes including *TNNI2* (Fig. 6b), *MYOD1* (Fig. 6c), and *MYOG* (Fig. 6d) were upregulated. The upregulation of MYOG and MyHC was confirmed at the protein level as well (Fig. 6e). Taken together, the data clearly showed that modest depletion of EZH2 inhibits proliferation and promotes differentiation.

EZH2 has been shown to promote differentiation in ERMS^23^ and we sought to understand if EZH2 also regulated *TBX3* in these cells as well. shEZH2 constructs were transiently transfected into RD cells and we found that depletion of EZH2 upregulated *TBX3* mRNA and inhibited *TBX2* mRNA (Fig. 6f). As our EZH2 depletion results in RH30 differed from previously published work, we sought to understand if our shRNA approach could recapitulate what has been shown with an siRNA approach in RD cells^23^. RD cells were grown to confluence, transiently transfected with shEZH2, and subjected to differentiation conditions for 2 days prior to immunofluorescence with MyHC antibodies. We found that MyHC was observed by immunofluorescence (Fig. 6g), consistent with what was observed with a siRNA approach^23^. Parallel samples were analyzed for EZH2 depletion by mRNA expression (Fig. 6h). Stable cell lines expressing shEZH2 could not be recovered, suggesting that EZH2 is critical for cell survival in ERMS cells.

**Fig. 7:**
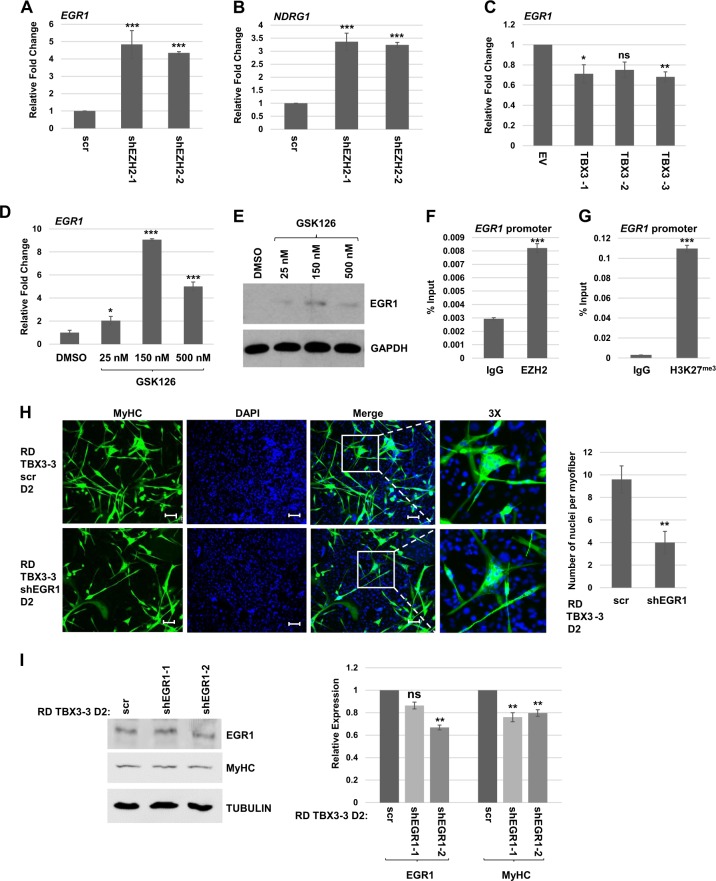
EGR1 is correlated with differentiation and repressed by EZH2 in RH30 cells. **a**, **b** mRNA expression of *EGR1* (**a**) and *NDRG1* (**b**) was assayed by qRT-PCR in RH30 shEZH2 and scr cell lines. Error bars, S.E. and ***p ≤ 0.001 versus scr. **c** TBX3 does not induce EGR1. mRNA expression of *EGR1* was assayed by qRT-PCR. Error bars, S.E. and *p ≤ 0.05, **p ≤ 0.01 versus scr. n.s. represents not statistically significant. **d**, **e** Inhibition of EZH2 induces EGR1. RH30 cells were treated with the indicated concentration of GSK126 or DMSO (vehicle control) for 72 h and harvested for RNA (**d**) and protein (**e**). Error bars, S.E. and *p ≤ 0.05, ***p ≤ 0.001 versus DMSO. **f** EZH2 binds the *EGR1* promoter. ChIP assays were performed on RH30 cells using antibodies against EZH2 and primers against the *EGR1* promoter. Error bars, S.E. and ***p ≤ 0.001 versus IgG. **g** The *EGR1* promoter contains H3K27^me3^. ChIP assays were performed on RH30 cells using antibodies against H3K27^me3^ and primers against the *EGR1* promoter. Error bars, S.E. and ***p ≤ 0.001 versus IgG. **h** Stable RD cell lines overexpressing TBX3 were transiently transfected with shEGR1 or scr when confluent and assayed by immunofluorescence for MyHC after 2 days of differentiation conditions (D2). DAPI was used to stain nuclei. Images were taken at ×100 and scale bars represent 100 μm. Nuclei per myofiber are quantitated in the right panel. Error bars, S.E. and **p ≤ 0.01 versus scr. **i** Cells as in (**h**) were analyzed for protein expression with the indicated antibodies and replicate blots are quantitated in the right panel. TUBULIN was used as the loading control. Error bars, S.E. and **p ≤ 0.01 versus scr. n.s. represents not statistically significant

Our results showed that depletion of EZH2 promotes differentiation in both ARMS and ERMS cells. However, overexpression of TBX3 only promoted differentiation in ERMS cells. Recently, we have shown that the early response growth factor, EGR1, is highly expressed in ERMS and weakly expressed in ARMS. Ectopic expression of EGR1 in RH30 cells promoted differentiation^28^. EGR1 has been shown to be repressed by PRC2 under the control of the SS18-SSX fusion protein in synovial sarcomas^29^. EGR1 is also a target of the PRC2 complex in acute myeloid leukemia^30^ and in chondrogenesis^31^. To determine if the expression of EGR1 was correlated with the results obtained here, we examined *EGR1* mRNA expression in RH30 cells depleted for EZH2 and found that *EGR1* mRNA was upregulated upon *EZH2* depletion (Fig. 7a). A target gene of EGR1, *NDRG1*, was accordingly upregulated as well (Fig. 7b). *EGR1* mRNA expression was also examined in RH30 cells expressing TBX3 and we saw no upregulation of *EGR1* mRNA (Fig. 7c), strongly suggesting that EGR1 is required to promote differentiation. We also found that inhibition of EZH2 activity with the chemical inhibitor GSK126 upregulated *EGR1* mRNA in RH30 cells (Fig. 7d) and this upregulation could be detected at the protein level as well (Fig. 7e). To confirm that *EGR1* is a direct target of EZH2 in RH30 cells, we performed ChIP analysis and found that EZH2 (Fig. 7f) and H3K27^me^ (Fig. 7g) were found on the *EGR1* promoter in these cells. We also asked if transient depletion of *EGR1* could inhibit differentiation in RD cells stably expressing ectopic TBX3. We found that transient depletion of EGR1 reduced the number and size of myofibers observed (Fig. 7h). While the depletion of EGR1 achieved by this approach was modest, quantification of the MyHC protein level showed that the downregulation of MyHC closely correlated with the depletion of EGR1 (Fig. 7i).

### The PRC2-TBX3-TBX2 genetic axis exists in normal skeletal muscle

**Fig. 8:**
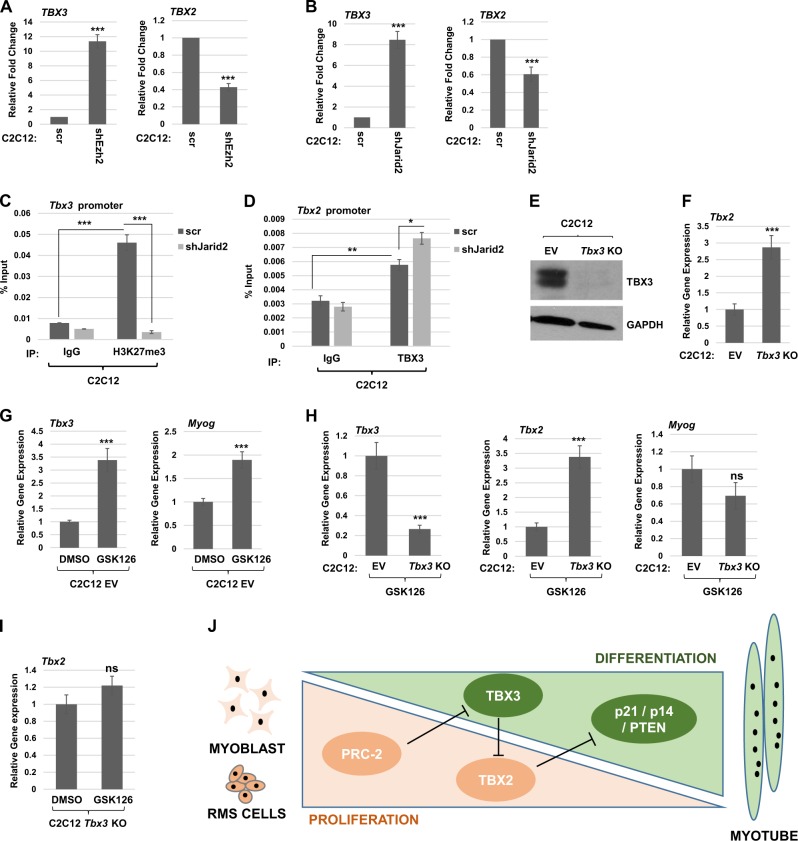
JARID2 and EZH2 directly repress TBX3 in skeletal muscle. **a**, **b** C2C12 cells were transfected with shEzh2 (**a**) or shJarid2 (**b**) and scr control. Stable isolates were assayed by qRT-PCR for mRNA expression of *Tbx2* and *Tbx3*. Error bars, S.E. and, ***p ≤ 0.001 versus scr. **c** Methylation of the *Tbx3* promoter is dependent on JARID2. ChIP assays were performed on C2C12 cell lines with scr or shJarid2 using antibodies against H3K27^me3^ and primers to the *Tbx3* promoter. Error bars S.E. and ***p ≤ 0.001 as indicated. **d** TBX3 binds the *Tbx2* promoter. ChIP assays were performed on C2C12 cell lines with scr or shJarid2 using antibodies against TBX3 and primers to the *Tbx2* promoter. Error bars S.E. and *p ≤ 0.05, **p ≤ 0.01 as indicated. **e** *Tbx3* KO cells analyzed by the western blot assay. Cells transfected with EV without *Tbx3* guide RNA are labeled EV. **f** *Tbx2* is upregulated upon *Tbx3* deletion as assayed by qRT-PCR. Error bars S.E. and ***p ≤ 0.001. **g** Cells transfected with EV without *Tbx3* guide RNA (EV) were treated with 1.25 μM GSK126 24 h post seeding for 48 h before assaying gene expression by qRT-PCR. Error bars S.E. and ***p ≤ 0.001 as indicated. **h** Inhibition of EZH2 does not lead to repression of *Tbx2* in *Tbx3* depleted cells. *Tbx3* KO cells and control (EV) were treated and assayed for gene expression as in (**g**). Error bars S.E. and ***p ≤ 0.001 versus EV. n.s. represents not statistically significant. **i** Inhibition of EZH2 does not alter *Tbx2* expression in *Tbx3* depleted cells. *Tbx3* KO cells were treated and assayed as in (**g**). n.s. represents not statistically significant. **j** Model of the PRC2-TBX3-TBX2 genetic axis in rhabdomyosarcoma and skeletal muscle. The PRC2 complex is highly expressed during proliferation in myoblasts and RMS (in orange) which represses TBX3 and allows TBX2 expression, which represses p21/p14/PTEN that are required for differentiation (in green). A decline in PRC2 expression derepresses TBX3 which represses TBX2 and promotes differentiation and myotube formation in skeletal muscle

To understand the relevance of these results in normal skeletal muscle, we used C2C12 cells depleted for JARID2 and EZH2 with shRNA constructs^32^ and assayed for the effect on *Tbx3* and *Tbx2* mRNA expression. We found that *Tbx3* mRNA was upregulated when EZH2 (Fig. 8a) or JARID2 (Fig. 8b) were depleted. *Tbx2* mRNA was correspondingly downregulated upon the depletion of EZH2 (Fig. 8a) or JARID2 (Fig. 8b). ChIP assays were used to assay for the presence of histone H3 lysine 27 trimethylation (H3K27^me3^) on the *Tbx3* promoter and we found that H3K27^me3^ was enriched on this promoter (Fig. 8c). Depletion of JARID2 resulted in a loss of this enrichment, indicating that H3K27^me3^ on the *Tbx3* promoter is dependent on JARID2 and the PRC2 complex (Fig. 8c). To determine if TBX3 directly repressed *TBX2* as we had seen in RH30 cells, we performed ChIP assays with antibodies against TBX3 and primers against the *Tbx2* promoter. We found that TBX3 binds the *Tbx2* promoter and that this binding is enriched upon depletion of JARID2, which induces TBX3 expression (Fig. 8d). To confirm the PRC2-TBX3-TBX2 axis in skeletal muscle, we ablated *Tbx3* in C2C12 cells using a CRISPR/Cas9 approach. Synthetic guides directed to the first and second exons of *Tbx3* were used to generate individual stable cell lines that were screened by genomic DNA analysis and protein expression by sequencing (data not shown) and western blot (Fig. 8e), respectively. The Cas9 containing plasmid without a guide sequence was used as the control (EV). *Tbx2* mRNA was assayed in the *Tbx3* KO cell line and we found that *Tbx2* was upregulated, confirming that TBX3 represses TBX2 (Fig. 8f). To confirm that EZH2 represses *Tbx3*, C2C12 EV cells were treated with the EZH2 inhibitor, GSK126. We found that *Tbx3* was upregulated when EZH2 was inhibited (Fig. 8g). As a positive control for GSK126, we also examined the expression of *Myog*, which has been shown to be upregulated immediately upon the depletion of EZH2^33^ and we found that *Myog* was upregulated (Fig. 8g). To confirm the requirement of TBX3 to mediate the EZH2 activation of TBX2, we treated EV and *Tbx3* KO cells with GSK126. As anticipated, *Tbx3* mRNA was not activated in the *Tbx3* KO cells upon EZH2 inhibition (Fig. 8h). TBX2 levels remained high in the *Tbx3* KO cells despite EZH2 inhibition, confirming that the loss of EZH2 is not sufficient to repress TBX2 in the absence of TBX3. The expression of *Myog* was also examined here and we saw no upregulation of *Myog* in the *Tbx3* KO cells (Fig. 8h). This result is further support for the high levels of TBX2 in the *Tbx3* KO cells as *Myog* is directly regulated by MYOD1 and we have shown that TBX2 profoundly inhibits MRF activity^27^. We also compared *Tbx2* expression in *Tbx3* KO cells treated with vehicle control or GSK126 and found no change in *Tbx2* mRNA expression (Fig. 8i), again confirming that TBX3 is required for the loss of EZH2 to lead to the repression of *Tbx2*.

## Discussion

The role of TBX3 in regulating the tumorigenesis of RMS was unknown before our work. We show that TBX3 acts to repress TBX2 in both RMS and normal muscle. TBX3 does not appear to promote oncogenesis of RMS cells, unlike what has been observed in melanoma and breast cancer cells^14,17^. Our results reveal the novel genetic axis of the PRC2 complex, TBX3, and TBX2 (Fig. 8j). JARID2 recruits the PRC2 complex to repress TBX3, which then relieves the TBX3 repression of TBX2, allowing TBX2 expression. In normal skeletal muscle, where the PRC2 complex is highly expressed in muscle precursor cells and sharply downregulated upon differentiation^19^, this maintains repression of the antiproliferative TBX3 and expression of the proproliferative TBX2 when PRC2 is expressed. In RMS, deregulated PRC2 activity leads to constitutive expression of TBX2 which functions to drive proliferation by direct repression of *CDKN1A*, *CDKN2A*, and *PTEN* while inhibiting the activity of MYOD1 and MYOG^27^.

The repression of *TBX3* by the PRC2 complex has been previously observed^34^. Genome wide profiling of a PRC2 component, SUZ12, and histone H3 trimethylated at lysine 27 (H3K27^me3^), identified a group of developmental regulator genes that are repressed by PRC2 to maintain pluripotency and thus poised for activation during ES cell differentiation and *TBX3* was included in this group of developmental regulators^34^. These results are consistent with what we observe in this study.

TBX3 had previously been shown to represses TBX2 expression by directly associating with the TBX2 promoter in a breast cancer cell line, MCF-12A^18^. However, unlike what has been observed in those previous studies which showed that TBX3 has oncogenic potential, we found no evidence that TBX3 promotes oncogenesis in RMS. Instead, we found that TBX3 acts as a tumor suppressor in RMS, largely due to its repression of TBX2.

Upregulation of the PRC2 complex has been shown be correlated with many cancer types^35^. JARID2, a noncatalytic, substoichiometric component of the PRC2 complex, has been shown to be upregulated in RMS, where it promotes cell viability and inhibits differentiation^26^. The catalytic subunit of EZH2 has been shown to be highly expressed in ERMS and siRNA-mediated depletion or pharmacological inhibition of EZH2 inhibits proliferation and induce differentiation of ERMS^23^. EZH2 was also shown to be required for viability of ARMS by repressing *FBX032*^24^. Treatment of RH30 or RD cells with siEZH2 leads to robust transient depletion of EZH2 after 24 h and induced apoptosis but did not induce *MYOGENIN* or *MYOD1*. Transient inhibition of EZH2 has been shown to induce *MYOGENIN* in skeletal muscle cells^33,36–38^. Our lab has recently shown that transient depletion of *EZH2* or *JARID2* does result in transient upregulation of *MYOGENIN* and *MYOD1* as others have seen, but sustained depletion of either factor leads to a block in Wnt signaling, which inhibits *MYOD1* and blocks differentiation^32^. RMS cells are deregulated in Wnt signaling^39^, so a similar mechanism is not anticipated in RMS. Here, we found that RH30 cells with a modest sustained depletion of *EZH2* generated by stable selection of a shRNA construct against *EZH2* led to a decrease in proliferation and an induction of differentiation, including the upregulation of MYOD1 and MYOG.

We show here that EZH2 represses *TBX3*, which allows the expression of the potent oncogene TBX2. This genetic axis also acts in skeletal muscle and provides a molecular explanation of previous results from our lab. We have shown that TBX2 is expressed in proliferating myoblasts and is sharply downregulated upon differentiation, while TBX3 is expressed throughout myogenesis and upregulated during differentiation^11^. Here, we establish that the down regulation of EZH2 upon skeletal muscle differentiation leads to the upregulation of TBX3 and the subsequent downregulation of TBX2.

Our results also showed that TBX3 is not sufficient to induce myogenic differentiation in ARMS, while depletion of EZH2 is sufficient to promote differentiation. These results were correlated with the expression of EGR1, which we have shown acts as a tumor suppressor in RMS that can induce differentiation in ARMS upon ectopic expression^28^. We found that *EGR1* is a target of EZH2 repression. EGR1 has also been shown to be a target of the PAX-FOXO1 fusion that characterizes ARMS^40^. Thus, EGR1 is both repressed by EZH2 and destabilized by PAX3-FOXO1 in ARMS. It remains to be determined if *ERG1* is a target of PRC2 in ERMS cells, but it may be that the lack of PAX3-FOXO1 in these cells is sufficient to allow EGR1 expression. Consistent with this hypothesis, we observed that the rescue of EGR1 expression by EZH2 by inhibition or depletion was relatively modest when compared to the level of EGR1 present in ERMS cells.

The PRC2-TBX3-TBX2 axis discovered in this work has significant implications for both RMS and skeletal muscle. The PRC2 complex serves as a central mediator between proliferation and differentiation and understanding the functions of its many downstream targets offers new insight into how skeletal muscle grows and repairs. TBX2 serves as a potent oncogene in RMS and many other cancers when overexpressed and thus, understanding the deregulation of TBX2 through the PRC2-TBX3-TBX2 axis offers insight in new approaches to inhibit tumor growth of RMS and additional cancers. This work also provides a mechanistic understanding of the effect of PRC2 in RMS and offers additional support to the therapeutic value of PRC2 inhibition in RMS.

## Materials and methods

### Cell culture

RD and RH30 cells (ATCC) were grown in Dulbecco’s modified Eagle medium (DMEM) supplemented with 10% fetal bovine serum (FBS) (Hyclone, Fisher Scientific, Waltham, MA, USA) according to standard protocols. RD2 and RH28 were obtained from Denis Guttridge, Medical University of South Carolina, and grown as described above. All cell lines were authenticated by Bio-Synthesis (Lewisville, TX, USA) using STR analysis on September 14, 2011. Cell lines are routinely screened for mycoplasma infection using the LookOut Mycoplasma quantitative polymerase chain reaction (qPCR) Detection Kit (Sigma, St. Louis, MO, USA). All cell lines in this study were found to be mycoplasma negative prior to the study and following the study. C2C12 myoblasts (ATCC, Manassas, VA, USA) were grown in DMEM supplemented with 10% FBS. To induce differentiation, cells were grown to 90% confluence and the media switched to DMEM supplemented with 2% horse serum (Hyclone, Fisher Scientific). Cells were grown in differentiation medium for the number of days indicated in each experiment.

### Drugs

GSK126 (Cayman Chemicals) was dissolved in DMSO at a concentration of 28.5 mM and 0.1% DMSO was used as the vehicle control. Blasticidin (Corning, Fisher Scientific) was dissolved in ddH_2_O at 10 mg/ml and used at 10 μg/ml in RH30 cells and 2 μg/ml in RD cells. Puromyocin (Corning, Fisher Scientific) was dissolved in ddH_2_O at 10 mg/ml and used at 2 μg/ml in all cell lines. Drugs were diluted in DMEM for use at the indicated concentrations.

### Plasmids

TBX3 was cloned into the pEF TOPO (Invitrogen, Thermo Fisher) vector as previously described^11^. pEF was used as the vector control and contains the gene for blasticidin resistance. shRNA against TBX2 and scr control were previously described^27^. shRNA constructs are in the pLKO.1 vector, which contains the gene for puromycin resistance.

### Cell transfection

Cells were transfected with the indicated plasmids using calcium phosphate according to standard protocols or Turbofect transfection reagent (Fisher Scientific) according to manufacturer’s protocol. Transient transfections were harvested at 48 h post transfection or as indicated. Stable cell lines were made by transfecting cells with linearized plasmids and selecting for drug resistant colonies. Glass rings were used to isolate individual clones. Cells were recovered by trypsinization and transferred to single wells for propagation.

### Chromatin immunoprecipitation (ChIP)

ChIP assays were performed and quantified as described previously^41^ with the following modifications: A total of 1 × 10^7^ cells were used for each immunoprecipitation and protein A agarose beads (Life Technologies, Carlsbad, CA, USA) were used to immunoprecipitate the antibody–antigen complexes. Primers are described in Supplemental Table 1 and the antibodies used are listed in Supplemental Table 2. The real time PCR was performed in triplicate. The results are represented as percentage of IP over input signal (% Input). All ChIP assays shown are representative of four independent experiments. Standard error from the mean was calculated and plotted as error bar.

### Immunofluorescence

Cells were grown on cover slips, fixed with paraformaldehyde, blocked with 10% goat serum, 1.0 % NP-40 in phosphate buffered saline (PBS) for 1 h and washed with PBS. Primary antibodies against MyHC(MF20, Developmental Studies Hybridoma Bank, Iowa City, IA, USA) were incubated overnight at 4 °C, washed with PBS, and detected by Alexa Fluor-488 goat anti-mouse antibody (Life Technologies). Cell nuclei were stained by incubating with 1 μM DAPI (Life Technologies) for 5 min.

### Proliferation assay

A total of 2 × 10^4^ cells per well were seeded in six-well plates and on the indicated days, cells were counted under a light microscope using a hemocytometer. Cell viability was determined by trypan blue staining. Cell counting was performed in duplicate for four blinded biological replicates.

### Quantitative real time PCR (qRT-PCR)

Trizol (Life Technologies) was used for RNA extraction from cells. Two micrograms of total RNA was treated with DNase (Promega, Madison, WI, USA) and reverse transcribed with MultiScribe MuLV reverse transcriptase (Life Technologies). Forty nanograms cDNA was used for qPCR amplification (Life Technologies) with SYBR green PCR master mix (Life Technologies). Negative controls included no RT samples where no reverse transcriptase was added for each RNA sample. All quantitative RT-PCR (qRT-PCR) was performed in triplicate and three independent RNA samples were assayed for each time point. qRT-PCR data were calculated using the comparative Ct method (Life Technologies). Standard deviations from the mean of the [Δ] Ct values were calculated from three independent RNA samples. Primers used are listed in Supplemental Table 1. Where possible, intron spanning primers were used. qRT-PCR gene expression data are shown using the format of relative gene expression (Relative Fold Change). A fold change was calculated for each sample pair and then normalized to the fold change observed at *HPRT* and/or *18s rRNA*.

### Scratch assay

Cell mobility was assayed by scraping a straight line with a 20 µl pipet tip on a monolayer of cells grown to 100% confluency. Cell debris was removed by washing. To obtain the same field during the image acquisition, marks were created near the scratch line. The plate was placed in a CO_2_ incubator at 37 °C for the indicated hours. Percentage migration rates were calculated using following formula:

$${\mathrm{\% }}\,{\mathrm{Migration}}\,{\mathrm{rate}} = \left[ {{\mathrm{A}}_{\mathrm{0}}{\mathrm{ - A}}_{\mathrm{t}}} \right]{\mathrm{ / A}}_{\mathrm{0}} \ast \,{\mathrm{100}}$$

where “A_0_” is the area of the scratch immediately after the scratch was made, and “A_t_” is the area of the scratch after indicated time. Scratch area was measured using ImageJ software (NIH). All scratch assays shown are representative of four independent experiments.

### Soft agar assay

Soft agar assays were carried out in 60 mm culture dishes in which 2 ml of 0.7% Noble agar (Fisher Scientific) in 1× DMEM with 10% FBS was overlaid with 2 ml of 0.35% agar in 1× DMEM with 10% FBS containing the cells. Cells of each clone (3 × 10^5^) were plated in triplicate. One milliliter of culture medium was added to the top of each plate every 5 days and cells were grown at 37 °C in a CO_2_ incubator for 30 days. The plates were stained with 1 ml of 0.05% Crystal Violet (Fisher Scientific) for >1 h and colonies were counted using a dissecting microscope. All assays shown are representative of four independent experiments.

### Western blot

Cell extracts were made by lysing PBS washed cell pellets in the radioimmunoprecipitation assay buffer supplemented with protease inhibitors (Complete protease inhibitor, Roche Diagnostics, Indianapolis, IN, USA) and clear lysates obtained by centrifugation. Protein concentration was determined by the Bradford’s assay (Bio-Rad, Hercules, CA, USA) and 50 µg of protein was loaded for each well of sodium dodecyl sulfate polyacrylamide gel electrophoresis. Resolved proteins were then transferred onto a PVDF membrane using a tank blotter (Bio-Rad). Membranes were blocked with 5% milk in 1× Tris-buffered saline plus Tween 20 (TBST) and followed by incubation with primary antibody overnight at 4 °C. After washing membranes with 1× TBST, membranes were incubated with the corresponding secondary antibody, washed with 1× TBST and incubated with chemiluminescent substrate according to the manufacture’s protocol (Pierce SuperSignal, Fisher Scientific) and visualized by autoradiography and/or an iBright FL1000 Imager (Invitrogen, Thermo Fisher Scientific, Waltham, MA, USA). Blots were quantitated using ImageJ software (NIH) or iBright Analysis software. Four biological replicates were performed for each western blot assay.

### Genome editing of *Tbx3* gene

Guide sequences for the *Tbx3* gene were chosen using an online CRISPR Design Tool (http://tools.genome-engineering.org). Guide containing DNA oligonucleotides was designed and cloned into pSpCas9(BB)-2A-Puro (PX459) V2.0 as described^42^. pSpCas9(BB)-2A-Puro (PX459) V2.0 was a gift from Feng Zhang (Addgene plasmid #62987; http://n2t.net/addgene:62987; RRID:Addgene_62987). Following selection of individual puromycin resistant clones, genomic DNA was isolated using Trizol (Invitrogen, Thermo Fisher Scientific, Waltham, MA, USA) and PCR amplified targeted regions were sequenced.

### The Cancer Genome Atlas (TCGA) sarcoma expression data analysis

Using cBioPortal (www.cbioportal.org), mRNA expression data for TBX3 and TBX2 in sarcoma patients (TCGA, Provisional, 265) were visualized on a heatmap and clustered. The mRNA expression Z-score (RNAseq V2 RSEM) was set to default (±2). For further analysis, mRNA expression (RNA Seq V2 RSEM) data of TBX3 and TBX2 for sarcoma patients were downloaded from TCGA (https://cancergenome.nih.gov/) database using GDAC Firehose. To better understand the correlation between the expression of TBX3 and TBX2 in these patients, two cohorts of the top one-third TBX3 and TBX2 expressing samples were combined and duplicates samples were removed (*N* = 113). A correlation analysis was performed, a Spearman correlation coefficient (*r*_*s*_) was calculated and a scatter plot was generated for TBX3 and TBX2 expression using GraphPad Prism 8.0.

### Statistics

Data are presented as means ± standard errors. Statistical comparisons were performed using unpaired two-tailed Student’s *t*-tests. Spearman correlation coefficient (*r*_*s*_) was calculated for TBX3 and TBX2 mRNA expression in TCGA samples using GraphPad Prism 8.0. Statistical significance are denoted by asterisks as indicated in figures with “***”, “**”, and “*” for *p*-value ≤ 0.001, 0.01, and 0.05, respectively.

## Supplementary information

Supplemental Table 1

Supplemental Table 2

Supplemental File
