# Supplementary material for: TBX3 represses TBX2 under the control of the PRC2 complex in skeletal muscle and rhabdomyosarcoma
Source: Oncogenesis. 2019 Apr 12;8(4):27. doi: 10.1038/s41389-019-0137-z (PMC6461654; doi:10.1038/s41389-019-0137-z)
Supplement: Supplementary file 1 — Supplemental Table 1 [file 41389_2019_137_MOESM1_ESM.docx]

**Supplemental Table 1**

Oligonucleotides used in study: H indicates human specificity, M indicates murine specificity and M/H indicates both.

**Quantitative Real Time PCR (qRT-PCR):**

HPRT1 F: 5’ TGACACTGGCAAAACAATGCA 3'

m/h R: 5’ GGTCCTTTTCACCAGCAAGCT 3'

18S rRNA F: 5’ CGCCGCTAGAGGTGAAATTCT 3'

M/H R: 5’ CGAACCTCCGACTTTCGTTCT 3'

TBX2 F: 5’ GAGGACGAGGTGGAGGAC 3'

m/h R: 5’ GCTGACTCGCACCTTGAAG 3'

CDKN2A(p14) F: 5’ ATGCTACTGAGGAGCCAGC 3'

H R: 5’ ACCAGCGTGTCCAGGAAG 3'

CDKN1A (p21) F: 5’ GGAACTTCGACTTTGTCACC 3'

H R: 5’ CAGTGACAGGTCCACATGG 3'

PTEN F: 5’ GCTATGGGATTTCCTGCAG 3'

m/h R: 5’ CTAGCTGTGGTGGGTTATGG 3'

TNNI2 F: 5’ CAGCACCTGAAGAGTGTGATG 3'

H R: 5’ GGTAGTTCTGCTTCTCTGCCTC 3'

LMOD2 F: 5’ GTGGAAAGGTTGCAGAAGACA 3'

H R: 5’ TCGTCACTGTCTTCTTCCTCCT 3'

MYOD F: 5’ TGGAGCTACTGTCGCCAC 3'

H R: 5’ GCTCTTCGGGTTTCAGGAG 3'

Myogenin F: 5’ AGCATCACGGTGGAGGATATG 3'

H R: 5’ CAGTTGGGCATGGTTTCGT 3'

EZH2 F: 5’ CCATGTTTACAACTATCAACCCTG 3'

m/h R: 5’ ACTCTCGGACAGCCAGGTAG 3'

EGR1 F: 5’ CACCCCAGACCAGAAGC 3’

m/h R: 5’ GCTGGGTTTGATGAGCTG 3’

**Chromatin immunoprecipitation (ChIP) assay:**

TBX2 promoter F: 5’ TTCTGTTGACATGTTTTCTTAC 3'

m/h R: 5’ CTGTGACTATCTCACATGTCC 3'

TBX3 promoter F: 5’ TCAGAACCCAGTCTCTCGC 3'

H R: 5’ CACACTGGGACTGAAACACAC 3'

TBX3 promoter F: 5’ CAAAGGCTCTCAGGAGACAG 3’

M R: 5’ CACGCAAATGCATTCAAG 3’

EGR1 promoter F: 5’ GAGCAACCAGCTGCGAC 3’

H R: 5’ CCTTCTTCCCTCCTCCCA 3’

Chr19 F: 5’ TGGGAAAACTCTCCAGGAC 3’

H R: 5’ ctttggttgcctgtgctt 3’

IgH F: 5’ GCCGATCAGAACCAGAACACCTGC 3’

M R: 5’ TGGTGGGGCTGGACAGAGTGTTTC 3’

CRISPR:

sgTBX3m 1 top F: 5’ CACCGCTTCGCCATGAGCGCGGTAC 3’

sgTBX3m 1 bottom R: 5’ AAACgtaccgcgctcatggcgaagC 3’

sgTBX3m 2 top F: 5’ ccacgTCCATTGCTCCCCCGTAGCG 3’

sgTBX3m 2 bottom R: 5’ AAACcgctacgggggagcaatggaC 3’

TBX3 sg1 gDNA F F: 5’ CGGACTGGTTCCCTGTCT 3’

TBX3 sg1 gDNA R R: 5’ cagggatgagaaagggatg 3’
