# Supplementary material for: TBX3 represses TBX2 under the control of the PRC2 complex in skeletal muscle and rhabdomyosarcoma
Source: Oncogenesis. 2019 Apr 12;8(4):27. doi: 10.1038/s41389-019-0137-z (PMC6461654; doi:10.1038/s41389-019-0137-z)
Supplement: Supplementary file 2 — Supplemental Table 2 [file 41389_2019_137_MOESM2_ESM.docx]

**Supplemental Table 2**

Antibodies used in study

**Western blot analysis:**

**Antibody:** **Manufacturer:**

TBX2 (C-17) Santa Cruz Biotechnology (sc-17880)

TBX2 (D-3) Santa Cruz, Biotechnology (sc-514291)

TBX3 (A-20) Santa Cruz, Biotechnology (sc-17817)

TBX3 ABclonal (A4144)

GAPDH (MAB374) Millipore

EZH2 (D2C9) Cell Signaling

Myosin Heavy Chain (MF20) Developmental Studies Hybridoma Bank

Myogenin (F5D) Developmental Studies Hybridoma Bank

V5 (MA5-15253) Piece, Thermofisher

**Chromatin Immunoprecipitation Assay:**

**Antibody:** **Manufacturer:**

TBX3 ABclonal (A4144)

EZH2 (D2C9) Cell Signaling

H3K27^me3^ Genetex

normal mouse IgG Santa Cruz Biotechnology (sc-2025)

normal rabbir IgG Santa Cruz Biotechnology (sc-2027)
